# Association between daytime napping and cognitive impairment among Chinese older population: a cross-sectional study

**DOI:** 10.1265/ehpm.23-00031

**Published:** 2023-11-22

**Authors:** Hong Zhang, Lijuan Zhang, Chen Chen, Xiaoni Zhong

**Affiliations:** Department of Health Statistics, School of Public Health, Chongqing Medical University, Chongqing 400016, PR China

**Keywords:** Cognitive impairment, Daytime napping, Cross-sectional study, The restricted cubic spline

## Abstract

**Background:**

Both napping and nighttime sleep duration have been reported to be associated with cognitive function in older adults, whereas little is known about the association between daytime napping and cognitive impairment in different nighttime sleep duration subgroups. This study aimed to explore the correlation between daytime napping and cognitive impairment across nighttime sleep duration subgroups.

**Methods:**

A cross-sectional study was conducted by using the fourth survey of China Health and Retirement Longitudinal Study (CHARLS). We utilized the Mini-Mental State Examination (MMSE) scale to define cognitive impairment, and the daytime napping and nighttime sleep duration was self-reported by individuals. We applied the Restricted Cubic Spline (RCS) to analysis the dose-response relationships between daytime napping and cognitive impairment. And the multivariate Logistic Regression Model (LRM) was performed to evaluate the association of daytime napping and cognitive impairment.

**Results:**

A total of 3,052 individuals were included, of which 769 were cognitive impairment. The RCS showed there were non-linear association between daytime napping and cognitive impairment in all participants group and longer nighttime sleep duration subgroup (*P_Non-linear_* < 0.05, *P_Daytime napping_* < 0.05). The LRM revealed no napping (OR = 1.62, 95%CI 1.14–2.30) and excessive napping (1.64 95%CI 1.09–2.48) were related to cognitive impairment in longer nighttime sleep duration subgroup.

**Conclusions:**

Daytime napping had nonlinear association with cognitive impairment in Chinese elderly population. No napping and excessive daytime napping (>90 minutes) were related to cognitive impairment in participants with 7 and more hours nighttime sleep duration.

**Supplementary information:**

The online version contains supplementary material available at https://doi.org/10.1265/ehpm.23-00031.

## 1. Background

Cognitive impairment is the transition status between normal aging and dementia, and mainly presented as declines in memory, attention and cognitive function [1]. Currently, more than 55 million people live with dementia worldwide, and there are nearly 10 million new cases every year (https://www.who.int/news-room/fact-sheets/detail/dementia). A study showed the conversion rate from mild cognitive impairment to Alzheimer’s disease was 10%–15% per year [2], indicating that nearly 100 million people were suffering from cognitive impairment across the globe each year. In China, the overall prevalence of dementia was 6.0% [3], and there would be 27.29 million people affected by dementia in 2030, the total cost would reach $812.42 billion [4]. To date, there are no pharmacologic treatments for most types of dementia, such as Alzheimer disease [5]. To prevent the development of dementia, more attentions should be paid to the early stage of the pathological process [6]. Therefore, it is imperative to identify the potential risk factors for cognitive impairment to help slow the progression of dementia.

Studies demonstrated that the risk factors for cognitive function were multifaceted, including age, sex, education, hypertension, depression and so on [7–10]. Among them, sleep duration is an important risk factor for cognitive decline [11–13]. A study based on nationwide and prospective cohort of Korean elderly showed sleep-related parameters like mid-sleep time, sleep duration, and latency were related to cognitive decline [14]. Another international cross-sectional study conducted in 67 sites in the United States, Canada, Australia, and Japan testified shorter sleep duration was linearly associated with higher Amyloid-β burden, which was the early pathological change of Alzheimer disease, and long sleep duration was associated with worse performance across multiple cognitive domains [15]. In China, the associations between sleep and cognitive function have also been explored. Li’s research demonstrated that people with longer sleep duration, compared to normal group (usually 7 hours per day), had lower mental status scores and lower memory scores [9]. And in Xu’s study, sleep conditions or parameters such as insomnia, daytime dysfunction, and excessive time in bed, were linked to higher risk of all-cause cognitive disorders [11]. Zhang’s research demonstrated that an inverted U-shaped relationship exists in sleep duration and global cognition, with the highest cognitive scores observed for sleep duration between 6 and 9 hours [16].

Recently, attention has been paid on the relationship between daytime napping and cognitive function, and long nap duration was significantly associated with high risk of cognitive frailty among the older adults in nursing homes [17]. In China, napping is very common and is believed to help make up for lack of nighttime sleep and improve health status [18, 19]. Yin’s research reported that about 44.7% of people had the habit of napping in the 60–69 age group in China [20]. A cohort study conducted in Zhejiang Province in eastern China investigated 10,740 participants aged over 60 years, and found compared to participants with no more than 30 minutes of post-lunch napping, that those who did not nap or napped longer had significantly higher risks for cognitive impairment [21]. However, researches above only included participants in partial sections of China, and little is known about the association between daytime napping and cognitive impairment in different nighttime sleep duration subgroups. Furthermore, the dose-response relationship of daytime napping and cognitive impairment is rarely mentioned. Therefore, based on the China Health and Retirement Longitudinal Study (CHARLS), we grouped nighttime sleep duration by the dose-response relationship analysis, and investigated the association between napping and cognitive impairment across nighttime sleep duration subgroups.

## 2. Materials and methods

### 2.1. Study population

**Fig. 1:**
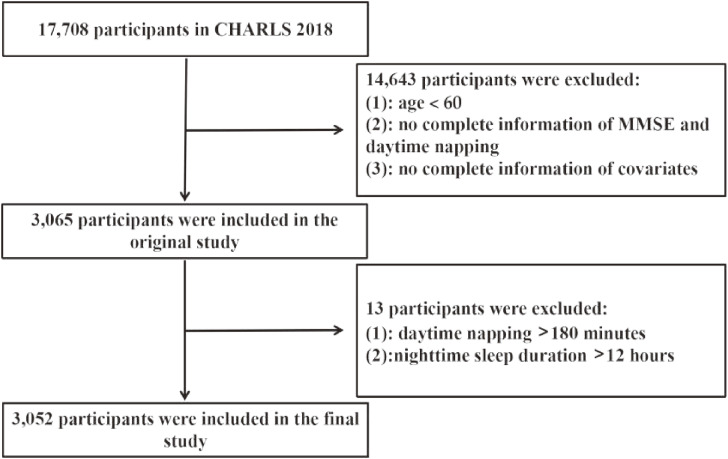
Flowchart of study participants.

This study was based on the fourth survey of CHARLS. It started the baseline survey in 2011, followed by three tracking surveys in 2013, 2015, and 2018. And it’s a national population-based survey of community-dwelling, adults aged 45 years and older, including 10,287 households, 17,708 individuals, and covering 150 counties in 28 provinces [22]. In this study, we formulated the inclusion criteria for the study subjects: age ≥60 years old, respondents with complete information on daytime napping, the Mini-Mental State Examination (MMSE) scale and other covariates. And we excluded the outliers: daytime napping >180 minutes and nighttime sleep duration >12 hours. Based on these criteria, 3,052 subjects were selected from 17,708 patients in the fourth CHARLS follow-up. (Fig. 1)

### 2.2. Cognitive impairment

The Mini-Mental State Examination (MMSE) is one of the commonly used scales for dementia and cognitive impairment screening, which has been proven to be reliable and valid for elderly Chinese [23, 24]. It includes seven aspects: cognitive function, orientation, reaction, attention, calculation, recall, and language. There are 30 questions in total, each answer correctly scored 1 point, and the total score range of the scale is 0–30 points. Participants were first categorized into cognitive impairment and normal according to their MMSE score and education. The cutoff value of MMSE was set at 18 for illiterate individuals, 21 for individuals with 1–6 years of education, and 25 for individuals with seven or more years of education, and cognitive impairment was defined as those whose MMSE score was lower than the cutoff value according to their education level [25].

### 2.3. Daytime napping and covariates

In CHARLS, daytime napping was measured by investigators asking subjects “During the past month, how long did you take a nap after lunch?” The unit was minutes, or 0 minutes if there was no nap. Nighttime sleep duration was collected by asking the similar question to participants, and the unit was hours. Other covariates included demographic characteristics such as age, sex, and education, lifestyle, and behaviors, such as smoking, alcohol consumption, health, and disease conditions such as hypertension, depression and dyslipidemia, and social welfare, such as medical insurance and pension. Investigations of depression in the CHARLS were largely done through the American Epidemiological Research Center’s 10-Item Scale (CESD-10), and the total score was between 0 to 30, and participants with more than 10 scores were considered to have depressed symptoms. All the covariates were reported associated with cognitive function [10, 26–28]. The variable coding rules are shown in Table S1 (see in Supplementary Material).

### 2.4. Statistical analysis

All the data in this study were obtained from the CHARLS database in DTA format, and imported into SAS version 9.4 and R Studio version 4.0 for analysis. We compared the characteristics of participants with Student’s t-test for normally distributed continuous variables, Wilcoxon rank sum test for skewed continuous variables, and Chi-square test for categorical variables. The dose-response relationships between sleep (daytime napping and nighttime sleep duration) and cognitive impairment were evaluated on a continuous scale by the Restricted Cubic Spline (RCS) with 3 knots. Then the continuous daytime napping and nighttime sleep duration were transformed to be categorical variables. Finally the multivariate Logistic Regression Model (LRM) was applied to examine the association between daytime napping and cognitive impairment in different nighttime sleep duration subgroups.

## 3. Results

### 3.1. Sample characteristics

Characteristics regarding 3,052 participants were shown in Table 1. In this study, 769 participants were defined as cognitive impairment, accounting for 25.20%. The median age was 67 years, and 66.42% of the participants was male. The median of MMSE score was lower in the cognitive impairment group than in the cognitively normal group (*P* < 0.05). Participants with cognitive impairment were more likely to be “not married”, live in rural areas, have 7 and more years of education, have no socializing activities in recent month, and be depressed. The proportions of health insurance and pension were lower in cognitive impairment group (*P* < 0.05). The median of daytime napping and nighttime sleep duration were 30 minutes and 6 hours respectively, and there were no significant difference between cognitive impairment and cognitive normal groups.

**Table 1:** Sample characteristics in the follow-up study (N = 3,052)

**Variables**	**Total**	**Cognitive** **impairment**	**Cognitive** **normal**	***P***
**N**	3,052	769	2,283	-
Age (P_50_(P_25_,P_75_))	67(63,71)	66(63,72)	67(63,71)	0.1542
Male (%)	2,027(66.42)	517(67.23)	1,510(66.14)	0.5802
Married (%)	2,639(86.47)	638(82.96)	2,001(87.65)	0.0010*
Rural (%)	1,780(58.32)	528(68.66)	1,252(54.84)	<0.0001*
Education (%)	-	-	-	<0.0001*
Illiterate	55(1.80)	16(2.08)	39(1.71)	-
1 to 6 years of education	1,355(44.40)	215(27.96)	1,140(49.93)	-
7 and more years of education	1,642(53.80)	538(69.96)	1,104(48.36)	-
Smoking (%)	1,700(55.70)	451(58.65)	1,249(54.71)	0.0572
Drinking (%)	1,303(42.69)	304(39.53)	999(43.76)	0.0404*
Socializing (%)	1,840(60.29)	423(55.01)	1,417(62.07)	0.0005*
Daytime napping (P_50_(P_25_,P_75_))	30(0,60)	40(0,60)	30(0,60)	0.2693
Nighttime sleep duration (P_50_(P_25_,P_75_))	6(5,7)	6(5,8)	6(5,7)	0.1620
Hypertension (Yes, %)	1,320(43.25)	316(41.09)	1,004(43.98)	0.1620
Diabetes (Yes, %)	516(16.91)	121(15.73)	395(17.30)	0.3160
Dyslipidemia (Yes, %)	936(30.67)	204(26.53)	732(32.06)	0.0040*
Depression (Yes, %)	852(27.92)	259(33.68)	593(25.97)	<0.0001*
Health insurance (Yes, %)	3,008(98.56)	750(97.53)	2,258(98.90)	0.0056*
Pension (Yes, %)	1,236(40.50)	244(31.73)	992(43.45)	<0.0001*
MMSE (P_50_(P_25_,P_75_))	25(23,27)	21(19,23)	26(25,28)	<0.0001*

* represents *P* < 0.05.

### 3.2. The dose-response relationship analysis

**Fig. 2:**
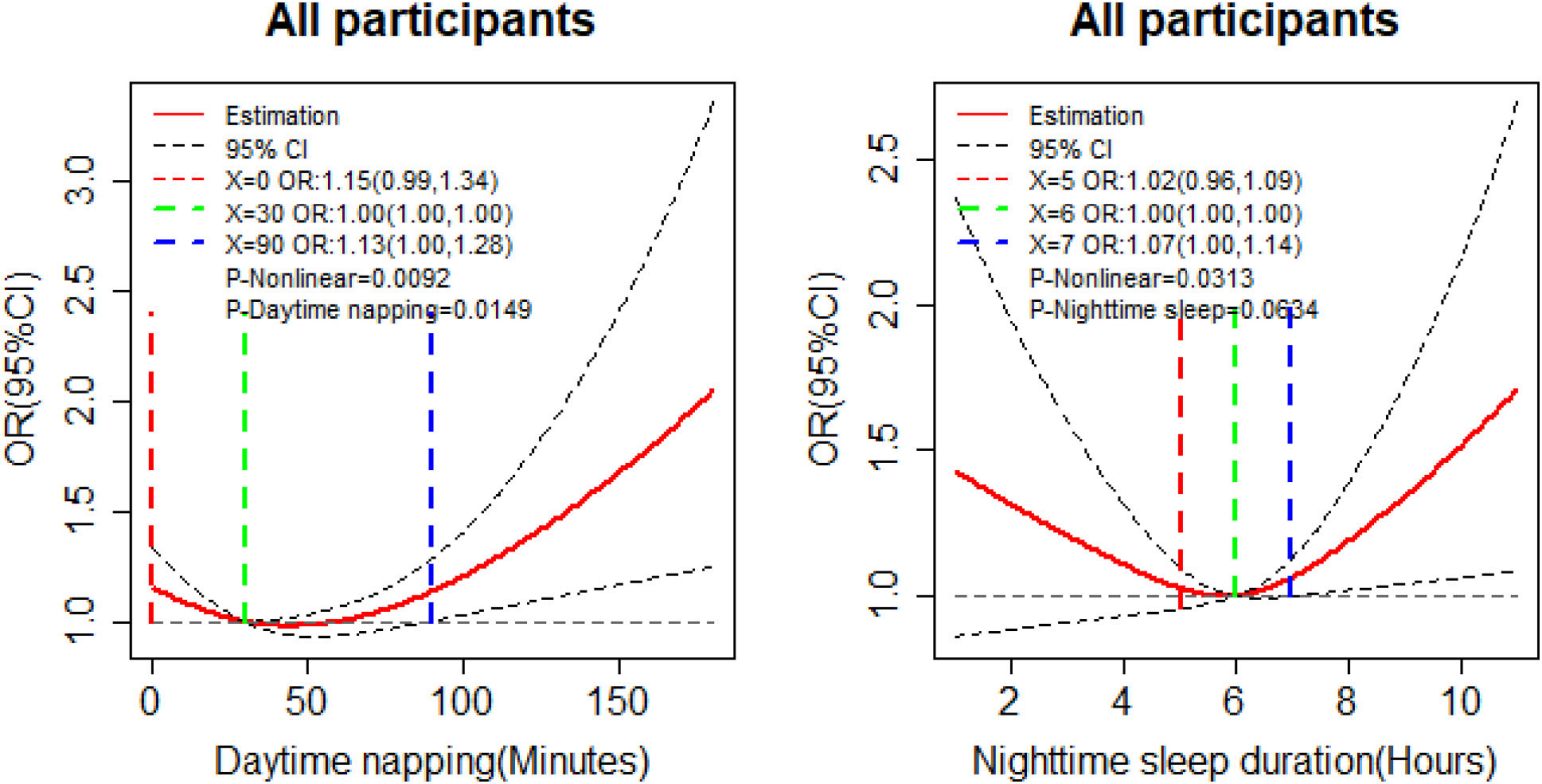
Dose-response relationships of daytime napping and nighttime sleep duration on cognitive impairment. Adjusted for age, sex, marital status, residence, education, smoking status, drinking status, social status, hypertension, diabetes, dyslipidemia, depression, health insurance, and pension.

As is shown in Fig. 2, daytime napping had a significant non-linear association with cognitive impairment (*P_Non-linear_* < 0.05, *P_Daytime napping_* < 0.05). The dose-relationship in daytime napping and cognitive impairment exhibited a J-shaped trend. The estimation odds ratio (OR) on no napping was 1.15 (95%CI: 0.99–1.34), then reduced until reach the lowest OR at reference value of daytime napping (30 minutes, OR = 1), and then increased thereafter. When daytime napping exceeded 90 minutes (OR = 1.13, 95%CI: 1.00–1.28), the lower limits of OR exceeded 1, which mean the risk of cognitive impairment among participants with more than 90 minutes would increase. Though the association between nighttime sleep duration and cognitive impairment was not significant (*P_Nighttime sleep_* > 0.05), and the dose-relationship of nighttime sleep duration and cognitive impairment showed a U-shaped trend (*P_Non-linear_* < 0.05). The reference value of sleep duration was 6 hours, which was the median value of all participants. And participants with more than 7 hours sleep (OR = 1.07, 95%CI: 1.00–1.14) increased the OR of cognitive impairment.

### 3.3. The RCS analysis in nighttime sleep subgroups

**Fig. 3:**
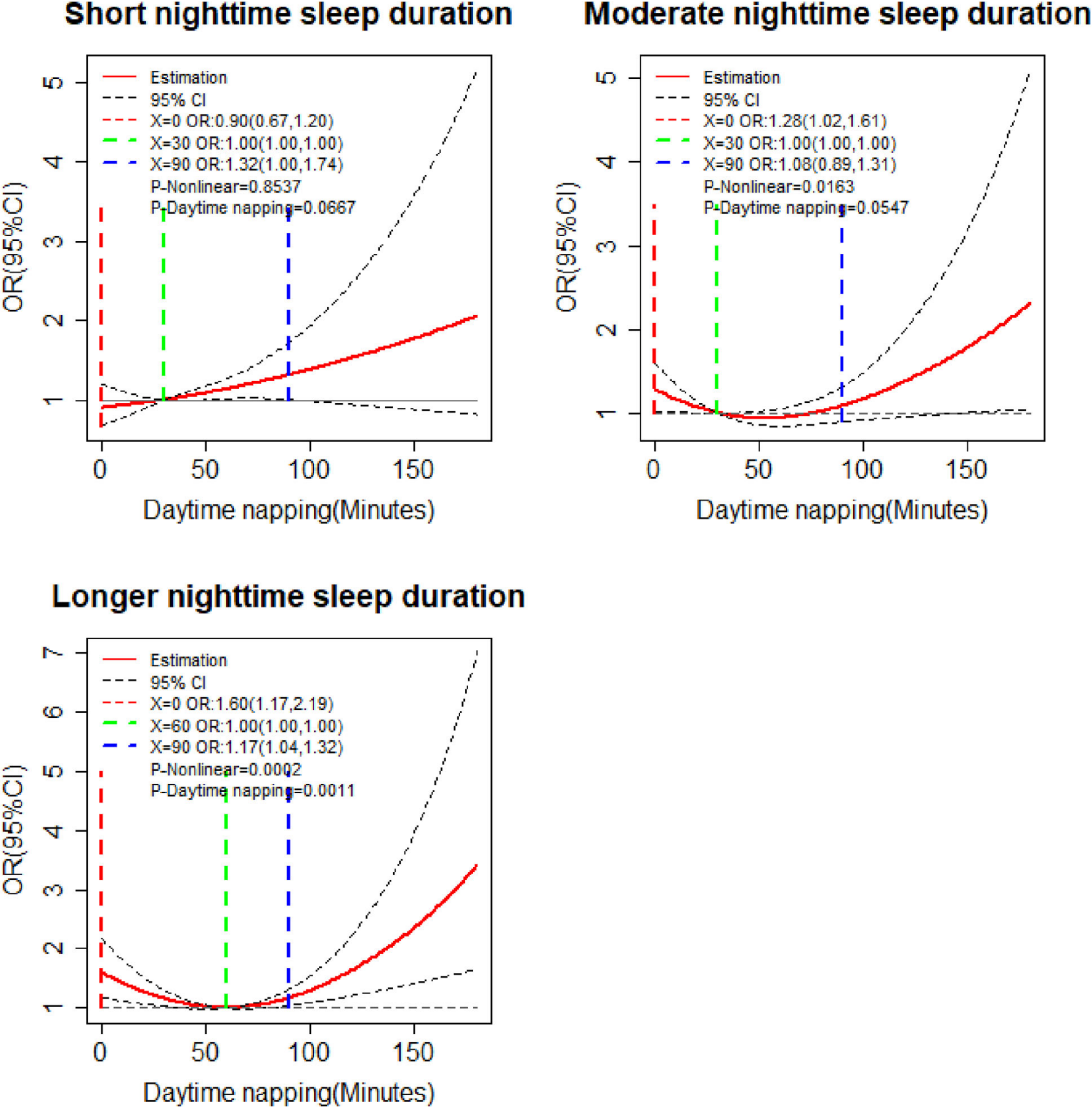
Dose-response relationships of daytime napping and cognitive impairment in different subgroups. Adjusted for age, sex, marital status, residence, education, smoking status, drinking status, social status, hypertension, diabetes, dyslipidemia, depression, health insurance, and pension.

Considering that participants with 6 hours sleep had the lowest OR of cognitive impairment in RCS analysis and the inter-quartile range of nighttime sleep duration, we divided nighttime sleep duration into three subgroups: short nighttime sleep duration (≤5 hours), moderate nighttime sleep duration (5–7 hours) and longer nighttime sleep duration (≥7 hours). The dose-response relationships between daytime napping and cognitive impairment in different subgroups were shown in Fig. 3. In the longer nighttime sleep duration subgroup, the relationship between daytime napping and cognitive impairment was nonlinear (*P_Non-linear_* < 0.05, *P_Daytime napping_* < 0.05). And the reference value of daytime napping was 60 minutes, participants with no napping (OR = 1.60, 95%CI: 1.17–2.19) or more than 90 minutes (OR = 1.17, 95%CI: 1.04–1.32), the OR of cognitive impairment increased. In the short and moderate nighttime sleep duration subgroups, no significant dose-response relationship between daytime napping and cognitive impairment have been found (*P_Daytime napping_* > 0.05).

### 3.4. The multivariate logistic regression model analysis

Giving the fact that lower risk for cognitive impairment in moderate daytime napping in the RCS analysis, combined with previous literature, we classified daytime napping to five groups: no napping (0 minutes), shorter napping (1–30 minutes), moderate napping (31–60 minutes), longer napping (61–90 minutes), excessive napping (>90 minutes), and moderate napping group was seen as the reference. The results of multivariate LRM were shown in Table 2. In all participants group, after adjusting covariates, daytime napping had no significant correlation to cognitive impairment. In shorter and moderate nighttime sleep group, no significant associations have been found between daytime napping and cognitive impairment also. Whereas in the longer sleep subgroup, the risk for cognitive impairment increased in no napping and excessive napping compared to the moderate napping (OR = 1.62, 95%CI 1.14–2.30, *P* < 0.05 in no napping, OR = 1.64 95%CI 1.09–2.48, *P* < 0.05 in excessive napping). A multicollinearity diagnosis was applied for the LRM (the adjusted model in all participants group), and the results showed that the VIF of all independent variables were less than 10, suggesting there was no obvious multicollinearity between the variables. (see in Table S2)

**Table 2:** The multivariate LRM in different sleep duration subgroups.

**Nighttime** **sleep Group**	**Daytime** **napping**	**Unadjusted model**		**Adjusted Model**	

		**OR(95%CI)**	***P***	**OR(95%CI)**	***P***
Reference	31–60	-	-	-	-
All participants N = 3052	0	1.15(0.93,1.41)	0.1952	1.12(0.90,1.39)	0.3128
	1–30	0.81(0.63,1.05)	0.1165	0.85(0.65,1.11)	0.2393
	61–90	1.14(0.80,1.61)	0.4542	1.06(0.73,1.53)	0.7505
	>90	1.46(1.12,1.91)	0.0050*	1.32(0.99,1.75)	0.0586
≤5 hours N = 940	0	0.80(0.56,1.16)	0.2350	0.77(0.52,1.13)	0.1754
	1–30	0.79(0.50,1.24)	0.3122	0.83(0.51,1.32)	0.4272
	61–90	1.46(0.73,2.82)	0.2730	1.35(0.66,2.71)	0.4013
	>90	1.30(0.76,2.19)	0.3380	1.27(0.72,2.24)	0.4029
5–7 hours N = 811	0	1.18(0.79,1.78)	0.4178	1.13(0.73,1.75)	0.5791
	1–30	0.60(0.35,1.01)	0.0573	0.60(0.34,1.03)	0.0672
	61–90	0.87(0.39,1.78)	0.7078	1.01(0.43,2.18)	0.9887
	>90	1.13(0.64,1.93)	0.6746	1.00(0.55,1.79)	0.9904
≥7 hours N = 1301	0	1.49(1.08,2.06)	0.0145*	1.62(1.14,2.30)	0.0068*
	1–30	0.98(0.65,1.46)	0.9283	1.05(0.68,1.60)	0.8346
	61–90	1.18(0.71,1.92)	0.5097	1.08(0.63,1.82)	0.7795
	>90	1.80(1.23,2.53)	0.0022*	1.64(1.09,2.48)	0.0180*

Adjusted model adjusted for age, gender, marital status, residence, education, smoking status, drinking status, social status, hypertension, diabetes, dyslipidemia, depression, health insurance, and pension. * represents *P* < 0.05.

## 4. Discussion

Using the nationally representative database, we evaluated the association between daytime napping and cognitive impairment. After adjusting potential covariates, results showed that no napping and excessive napping were associated with higher risk for cognitive impairment, highlighting the importance of modifiable behavior factors on the prevention of cognitive decline. Attentions should be paid on taking moderate daytime napping among elderly individuals with nighttime sleep duration over 7 hours, to help with cognitive function.

In this study, 769 participants were defined as cognitive impairment, accounting for 25.20%. It has also been observed that 24.8% of participants had cognitive impairment using the same standard as ours in Hunan Province [29] and that 25% of the participants in Brigola’s study scored lower than the cut-off point of MMSE for cognitive impairment [30], which is aligned with the present study. Furthermore, the prevalence of mild cognitive impairment in Jia’s research was 36.2% among participants aged over 55 years, and the reason for this discrepancy across studies may be due in part to the different standards for cognitive impairment [31].

A J-shaped relationship between daytime napping and cognitive impairment was observed in all participants group in the current study. In the short and moderate sleep duration subgroup, the nonlinear association was not significant. Compared to all participants group, the non-linear trend between daytime napping and cognitive impairment was more pronounced in longer sleep duration subgroup, participants with no napping or more than 90 minutes napping would increase the risk of cognitive impairment. And the results of multivariate LRM further confirmed the correlation that both no nappers and excessive nappers had higher risk of cognitive impairment.

No napping associated to cognitive function was reported in previous studies. Many researchers have reported that excessive daytime napping could lead to the cognitive decline in elderly people with or without dementia, though the definition of “excessive napping” was inconsistent. Li’s research indicated that excessive daytime napping could elevate the risk of Alzeimer’s dementia, and was associated with worse cognitive function [32]. In Fu’s study, the cognitive scores decreased gradually as the napping time increased, and moderates or excessive nappers (≥30 minutes) had significantly lower cognitive scores, compared to no habitual daytime napping group [33]. Leng’s report demonstrated that men who napped for 120 minutes or more per day compared with 30 minutes nap were 66% more likely to develop cognitive impairment [34]. The present study added further evidence to the view that excessive napping may be a potential risk factor for cognitive impairment. In Li’s research, moderate nappers had better overall cognition than non-nappers or extended nappers [35]. Souissi’s study confirmed that compared to no nap, a 30 minutes of nap opportunity was help to overcome the negative effect of partial-sleep-deprivation on cognitive performances [36]. In Ong’s report, participants who had a 90 minutes afternoon napping encoded word pairs better than a comparable group who stayed awake, and that may be attributive to the increases in hippocampal activation after napping [37].

Previous study confirmed both short sleep duration and longer sleep duration were associated with consistently lower cognitive scores [38, 39]. In Ma’s research, they used two nationally representative aging cohorts to analyse the association between self-reported sleep duration per night and global cognitive score, and they found a U-shape relationship in sleep and global cognitive function [40]. In current study, though we observed an U-shaped dose-response relationship in nighttime sleep duration and cognitive impairment, when the nighttime sleep duration less than 6 hours, the correlation in nighttime sleep duration and cognitive impairment was not significant. This difference may be due in part to the extent of cognitive function decline that dose not meet the criteria for cognitive impairment. Tworoger’ study analyzed a two-years cohort data from the US Nurses Health Study, and found no statistically significant association in sleep duration and cognitive decline, which is in consonance with current research [41].

Although there was a large number of literature showing that both napping and nocturnal sleep affected cognitive function in middle-aged and elderly people, few researchers have performed subgroup analysis across nocturnal sleep duration. In the current study, the association between napping and cognitive impairment were mainly happened in longer sleep duration subgroups. This finding does not suggest 7 hours and more sleep duration was the risk factor for cognitive function. Actually, in this study the relationship between nighttime sleep and cognitive impairment was not significant. And researches proved that a 7 hour sleep duration might be the best choice for adults. A study investigated in Japan, China, Singapore, and Korea found that sleep less than 7 hours or more than 7 hours was associated with an increased risk of all-cause mortality [42]. In Li’s research, a 7 hours sleep duration was the ideal station for white middle-aged and older people in European, and too much or too little sleep was associated with poorer cognitive performance and mental health [43]. In all, the cognitive function of participants with longer nighttime sleep duration (≥7 hours) may be more vulnerable to daytime napping, and further cohort evidences are needed for the results.

Multiple mechanisms have been proposed for how sleep could mediate the development of cognitive impairment, including inducing inflammation, decreasing clearance of neurotoxic metabolites and amyloid clearance, altering normal amyloid metabolism and so on, and yet researchers still have not reached a consensus conclusion [27, 44–46]. As to daytime napping, there was some evidence supporting the neuroprotective effect of nap. In Studte’ study, daytime napping could improve hippocampus-dependent associative memories for older adults [47], which may prevent the onset of cognitive impairment or dementia. However, little is known about the mechanisms of interaction effect of daytime napping and nighttime sleep duration on cognitive function. It’s worth exploring whether sleep duration and daytime napping both were the independent risk factors, or they were affected by the result of cognitive decline in elders. In general, the physiological mechanisms of sleep and napping as well as the interaction effects on the cognitive impairment remains further study.

The limitations of our work merit discussion. Firstly, information with regard to daytime napping and nighttime sleep duration were self-reported by the participants and its potential inaccuracy might bias the results. Secondly, other sleep characters like frequency of daytime napping, sleep quality and sleep apnea were not taken into consideration. Thirdly, this study only incorporated into 3,052 participants from 17,708 individuals, it may lead to a reduction of sample representativeness. Finally, the study was based on the cross-sectional study, and that’s the reason why we couldn’t draw a causal conclusion, for there is a possibility of reverse causality.

## 5. Conclusions

To sum up, the dose-relationship between daytime napping and cognitive impairment was nonlinear. No napping and excessive daytime napping (>90 minutes) would increased the risk of cognitive impairment among those elderly population with 7 and more hours nighttime sleep duration.

## Supplementary information

**Additional file 1:** **Supplementary Table 1** (Table S1. Variables coding rules). **Supplementary Table 2** (Table S2. The results of collinearity diagnosis).

## Declarations

### Availability of data and material

The CHARLS datasets can be downloaded at the CHARLS home page (http://charls.pku.edu.cn/en).
