# Supplementary material for: Association between daytime napping and cognitive impairment among Chinese older population: a cross-sectional study
Source: Environ Health Prev Med. 2023 Nov 22;28:72. doi: 10.1265/ehpm.23-00031 (PMC10685077; doi:10.1265/ehpm.23-00031)
Supplement: Supplementary file 1 — Additional file 1: Supplementary Table 1 (Table S1. Variables coding rules). Supplementary Table 2 (Table S2. The results of collinearity diagnosis). [file ehpm-28-072-s001.docx]

**Supplementary Material**

**Supplementary Table 1** (Table S1. Variables coding rules)

**Table S1**. Variables coding rules

| **Variables** | | **Coding rules** |
| --- | --- | --- |
| Demographic characteristics | Age（year） | Continuous variable |
|  | Gender | 1=Male，2=Female |
|  | Marital status | 1=Married，2=Others |
|  | Residence | 1=Rural，2=Others |
|  | Education | 0=Illiterate，1=1 to 6 years of education，2=7 and more years of education |
| Lifestyle and behaviors | Smoking | 1=Yes，0=No |
|  | Drinking | 1=Yes，0=No |
|  | Socializing | 1=Yes，0=No |
|  | Daytime napping | Continuous variable |
|  | Nighttime sleep Duration | Continuous variable |
| Diseases condition | Hypertension | 1=Yes，0=No |
|  | Diabetes | 1=Yes，0=No |
|  | Dyslipidemia | 1=Yes，0=No |
|  | Depress | 1=Yes，0=No |
| Social welfare | Health insurance | 1=Yes，0=No |
|  | Pension | 1=Yes，0=No |

**Supplementary Table 2** (Table S2. The results of collinearity diagnosis )

| **Variables** | **Df** | **VIF** |
| --- | --- | --- |
| Age（year） | 1 | 1.1949 |
| Gender | 1 | 2.1919 |
| Marital status | 1 | 1.1182 |
| Residence | 1 | 1.6917 |
| Education | 2 | 1.2667 |
| Smoking | 1 | 1.9541 |
| Drinking | 1 | 1.2048 |
| Socializing | 1 | 1.0577 |
| Daytime napping | 4 | 1.0630 |
| Hypertension | 1 | 1.1185 |
| Diabetes | 1 | 1.1100 |
| Dyslipidemia | 1 | 1.1914 |
| Depression | 1 | 1.0608 |
| Health insurance | 1 | 1.0226 |
| Pension | 1 | 1.7467 |
